# Dysphagia due to anterior cervical osteophytosis: case report

**DOI:** 10.1590/2317-1782/20212020435

**Published:** 2021-12-20

**Authors:** Mateus Morais Aires, Gabriela Marie Fukumoto, Sarah Lima Ribeiro, Leonardo Haddad, Eliézia Helena de Lima Alvarenga

**Affiliations:** 1 Departamento de Otorrinolaringologia e Cirurgia de Cabeça e Pescoço, Escola Paulista de Medicina, Universidade Federal de São Paulo – UNIFESP – São Paulo (SP), Brasil.; 2 Faculdade de Medicina de Ribeirão Preto, Universidade de São Paulo – USP – Ribeirão Preto (SP), Brasil.

**Keywords:** Deglutition Disorders, Osteophyte, Spinal Osteophytosis, Aged, Speech Therapy

## Abstract

Anterior cervical osteophytosis is a noninflammatory condition characterized by calcification or ossification of the anterolateral paravertebral ligaments of the cervical spine. It affects 20 to 30% of the elderly, being responsible for 1.6% of the identifiable etiologies of dysphagia in the senile population. In advanced states, dysphagia due to cervical osteophytosis can lead to complications such as malnutrition, weight loss and aspiration pneumonia. This study aims to alert to this diagnosis, enabling early treatment of the condition. The case of a 66-year-old male patient with choking dysphagia for solids and nasal food reflux for 1 year is reported. Fiberoptic Endoscopic Evaluation of Swallowing showed bulging of the posterior pharyngeal wall and, with solid food supply, restriction to the retroflexion of the epiglottis, nasal reflux of the food and a large amount of food residue on the lesion. Cervical spine Computed Tomography identified the presence of anterior cervical osteophytes between the C3 and C6 vertebrae, the largest with anteroposterior length of 12 millimeters, narrowing the air column at the level of the oro- and hypopharynx. The patient was adequately treated with swallowing therapy by speech-language pathologist. The initial treatment strategy for symptomatic osteophytosis should be conservative, usually with a good response to swallowing therapy. Although they are rarely implicated in the etiology of dysphagia, considering its high prevalence, it is important that otolaryngologists and speech-language pathologists are attentive to this diagnosis, allowing early and effective treatment for the assisted patient, better prognosis and fewer complications of oropharyngeal dysphagia in the elderly.

## INTRODUCTION

Swallowing is a synergistic, sequential and harmonic neuromuscular process, in which food is transported from the mouth to the stomach. Any disruption in the swallowing movement, before, during or after eating, can be defined as dysphagia^(1)^. Presbyphagia, in turn, is understood as a change in swallowing in healthy elderly people and it can be silent and progressively slower, as it occurs in parallel with the aging process. It may be associated with natural changes in the head and neck anatomy and the gradual decline of physiological and neurological functions, with a reduction in laryngeal elevation, in the opening of the upper esophageal sphincter and in peristaltic movements^(2)^. The health status of the elderly can be affected by some aggressor factor, causing loss of their delicate balance and of the mechanisms that compensate for the swallowing disorder, thus developing dysphagia. It is estimated that the prevalence of dysphagia in people over 60 years of age is of 27%^(3)^.

The loss of functional reserve (the body resilient capacity to adapt to physiological stress) makes the elderly population more susceptible to dysphagia^(1)^. Comorbidities and chronic diseases, use of medications, cognitive impairment after cerebrovascular events or diseases of the upper aerodigestive system, which are more prevalent among the elderly when compared to younger individuals, can impair the well-adapted presbyphagic function and lead to dysphagia and aspiration^(1)^.

Swallowing disorders have increasingly been recognized as emerging problems by health professionals, especially those dealing with the senile population. Recently, the European Society for Swallowing Disorders and the European Union Geriatric Medicine Society recognized dysphagia as a geriatric syndrome^(3)^. Its main complications are malnutrition, dehydration and respiratory affections such as aspiration pneumonia. Additionally, several studies have highlighted the negative social and psychological impact of dysphagia, such as displeasure with eating, anxiety, panic and social isolation, with an incidence as expressive as 55% of institutionalized elderly^(1,3)^.

The syndromic treatment of oropharyngeal dysphagia in the elderly is based on compensatory measures, such as adaptation of food consistency, posture correction and swallowing maneuvers. If a specific cause of dysphagia is identified, targeted etiological treatment can be performed, with a better prognosis for dysphagia resolution and recovering quality of life^(2)^.

Anterior cervical osteophytosis (ACO) is a noninflammatory condition characterized by calcification or ossification along the anterolateral paravertebral ligaments of the cervical spine. Cervical osteophytes are common in the elderly, with a prevalence of 20 to 30%, and may be a cause of dysphagia in this population. They are usually asymptomatic radiological findings or cause unspecific symptoms, such as limited cervical movement or local pain. If they are bulkier, they can compress the posterior wall of the pharynx or esophagus, leading to oropharyngeal dysphagia. In advanced stages, the elderly can suffer from malnutrition and weight loss due to dysphagia caused by cervical osteophytes^(4)^.

Cervical osteophytes are estimated to be responsible for 1.6% of the identifiable etiologies of dysphagia in elderly people^(5)^. Despite being a rare cause, it is important that it be researched, as it is a potentially curable etiology with adequate treatment, whether conservative or surgical. The earlier the treatment, the better the results, as there are fewer irreversible tissue changes caused by chronic exposure to osteophytes in the upper digestive tract, such as inflammation and fibrosis with a reduction in peristalsis^(6)^.

Since there are few cases of dysphagia secondary to ACO reported in the literature, this study aims to alert the multidisciplinary team that attend to the elderly with dysphagia to suspect this diagnosis. This would enable early treatment of the condition, better prognosis and lower incidence of oropharyngeal dysphagia complications in the elderly.

### CLINICAL CASE PRESENTATION

The presented patient signed the Informed Consent Form, and the other ethical principles were complied with, respecting Resolution 466/12 of the National Research Ethics Council. This case report was approved by the Research Ethics Committee of the Federal University of São Paulo (UNIFESP), under opinion number 4.455.365.

**Figure 1:**
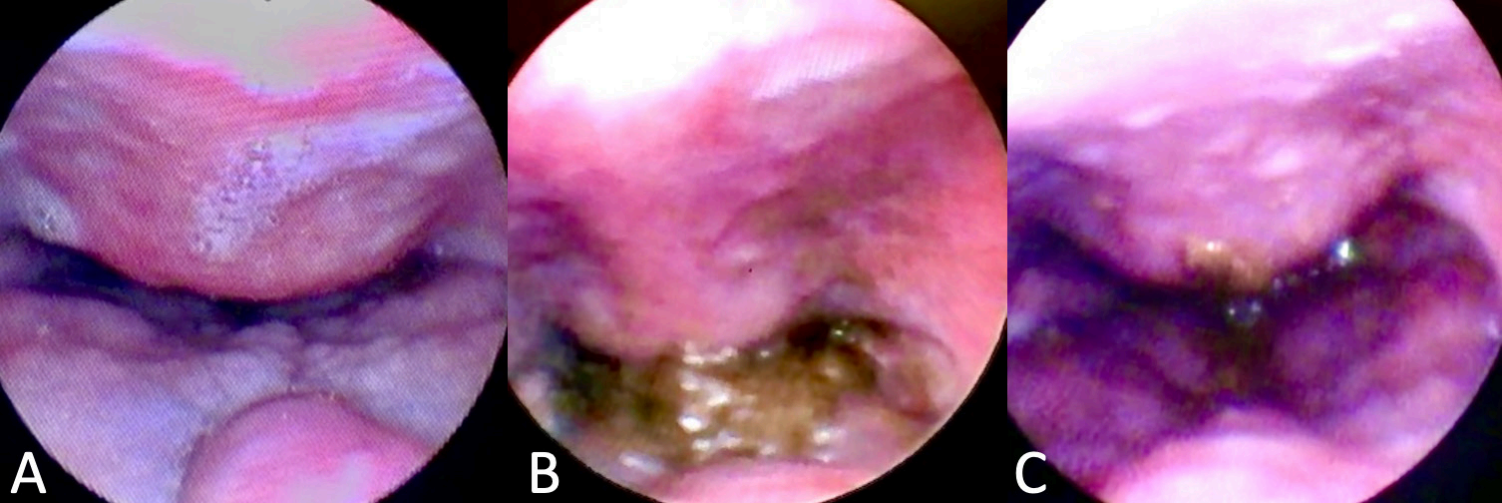
Fiberoptic Endoscopic Evaluation of Swallowing. Structural assessment demonstrating (A) spherical bulging of the posterior wall of the oropharynx, extending anteriorly towards the soft palate and base of the tongue and narrowing the aerodigestive pathway. Functional evaluation with solid consistency showing (B) nasal reflux of food and (C) ineffective clearance with food residue on the lesion after 3 swallows

**Figure 2:**
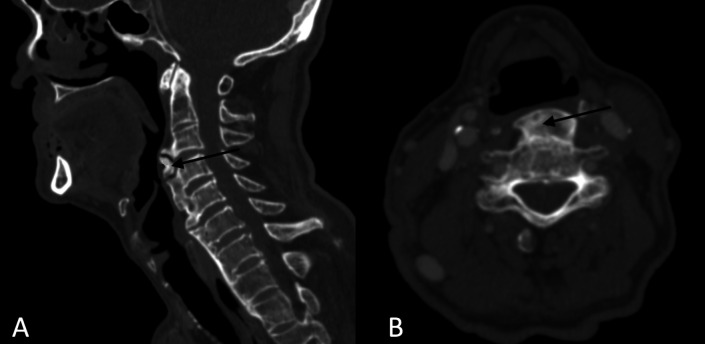
Neck computed tomography, sagittal (A) and axial (B) sections, showing cervical osteophytes from C3 to C6, the largest (arrow) measuring 12mm in the anteroposterior diameter

**Figure 3:**
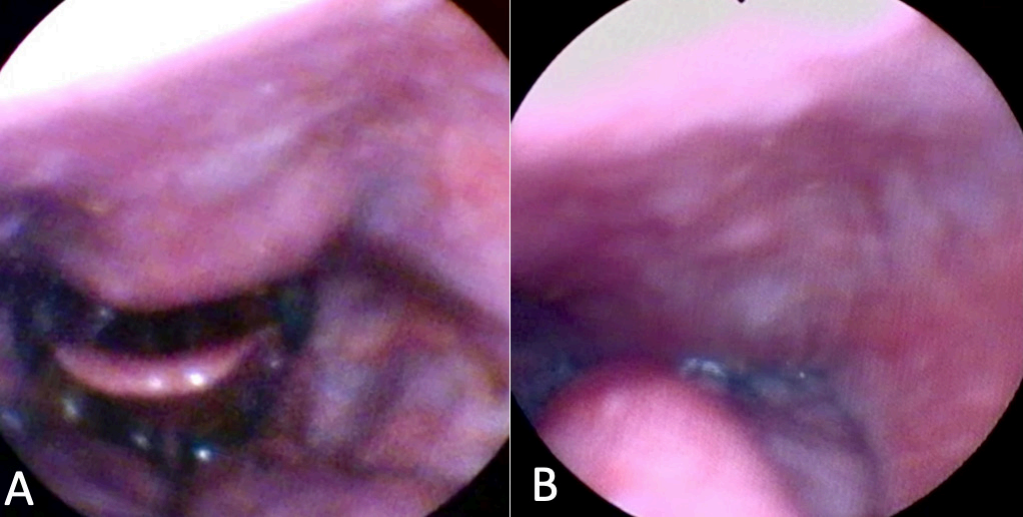
Fiberoptic Endoscopic Evaluation of Swallowing after speech therapy. Functional evaluation with chin tuck maneuver, (A) before swallowing, showing an increase in the pharyngeal and vallecular space by the maneuver, and (B) during swallowing, with a decrease in nasal reflux of food

A 66-year-old male patient attended an otorhinolaryngology appointment complaining of choking-type dysphagia for solids and nasal reflux of food for one year. He suffered a car accident 13 years ago and has diabetes mellitus. Fiberoptic Endoscopic Evaluation of Swallowing (FEES) was performed, with bulging of the posterior wall of the hypopharynx visualized in the structural evaluation, with salivary residue on the lesion (Figure 1). In the functional evaluation, after offering solid food, restriction to the retroflexion of the epiglottis, limitation of laryngeal elevation and nasal reflux of food, with a large amount of food residue above the lesion, in the posterior pharyngeal wall, was observed. In the postural maneuvers test, there was worsening of dysphagia on cervical extension and better clearance on head flexion. Computed tomography (CT) of the cervical spine was requested to assess the nature of the lesion, showed the presence of anterior cervical osteophytes between the C3 and C6 vertebrae, the largest with an anteroposterior length measuring 12 millimeters (mm), narrowing the air column at the ear level - and hypopharynx (Figure 2). Other causes of dysphagia were excluded, and the patient was treated with swallowing speech therapy and omeprazole, the latter aimed at pharyngolaryngeal reflux. Six individualized weekly speech therapy sessions were carried out, with the aim of compensating the swallowing mechanism when faced with a mechanical obstacle in the pharyngeal region. The main aspects worked were modification of the diet texture, avoiding solid and dry foods; postural swallowing maneuvers, tested during FEES, especially chin tuck (“head down” or “head flexion”), which increases the vallecula space (“opens the vallecula”) and improves the pharyngeal food transfer pressure; and tongue strengthening exercises to overcome the mechanical barrier of the oropharynx during food ejection. It was also recommended, when necessary, in view of the perception of stagnant food in the pharynx, alternating the offer of solid food with liquid. Repetition of the language exercises at home was recommended, 2 to 3 times a day. After the initial sessions, the patient showed a satisfactory response (Figure 3), maintaining quarterly follow-ups in the first year and every six months from the second year onwards. Referred to orthopedic assessment to discuss surgical feasibility, we opted for a conservative treatment due to good compensation with clinical measures. He has been under outpatient follow-up for 2 years, maintaining efficient swallowing, with no incidence of complications related to dysphagia. It was decided to maintain periodic reassessments, at least once a year, for early detection of a possible new imbalance in swallowing homeostasis related to the gradual decline of functions in the context of presbyphagia or the emergence of a new aggressive factor.

## DISCUSSION

Anterior cervical osteophytes are a common radiological finding in the elderly population, affecting 20 to 30% of patients over 60 years old^(7)^. Among its possible etiologies, idiopathic diffuse skeletal hyperostosis (Forestier's disease), ankylosing spondylitis, degenerative cervical spine disease and bone repair after trauma stand out. In addition to advanced age, male sex (6:1 compared to female) and obesity are well-established risk factors for the development of osteophytes^(5)^. According to a systematic review conducted by Verlaan et al.^(5)^, the most common site of osteophyte involvement is between the C3 and C5 vertebrae. Bony excrescence usually ranges from 1 to 2 mm, but can reach 30 mm. The reported patient is a typical osteophyte carrier, as he is a male, elderly, has a history of trauma and affects the C3 to C6 vertebrae.

Cervical osteophytes, even large, are often asymptomatic^(8)^. Chronic hyperostosis seems to be relatively well tolerated until a “trigger event”, such as asphyxia, aspiration or minor cervical trauma, causes an imbalance in the already borderline compensatory reserve, leading to a clinical picture. Multiple pathophysiological mechanisms have been described to explain osteophyte-related symptomatology, such as^(1)^ direct mass effect, causing mechanical obstruction or deviation of the pharynx or esophagus^(2)^; anchorage of the upper esophageal sphincter (UES) at the cricoid cartilage level^(3)^; restriction of laryngeal or epiglottis movement^(4)^; inflammation of the underlying soft tissue, leading to fibrosis and stenosis; and^(5)^ inflammatory neuropathy^(9)^.

The most common symptoms are reduced range of cervical movement and local pain. OCA rarely causes dysphagia. In a series of 116 patients with OCA, only 7 (6%) had dysphagia^(7)^. Studies show that the incidence of dysphagia secondary to OCA varies from 0.1 to 33%, depending on the definition of dysphagia and the diagnostic method^(7)^. On the other hand, dysphagia is rarely caused by cervical osteophytes. In a series of 3318 patients with dysphagia, only 55 (1.6%) had osteophytes, and more than half of these concomitantly had another etiology contributing to dysphagia^(10)^.

Osteophytes from the fifth and sixth cervical vertebrae (C5 and C6) are the most implicated in causing dysphagia. There is no linear correlation between osteophyte size and symptomatology, but it is known that osteophytes larger than 10mm significantly increase the risk of aspiration^(11)^. A recent cohort of 10 patients with OCA who are candidates for surgical treatment showed that half had weight loss and malnutrition due to dysphagia, confirming that the clinical picture can be severe^(4)^. A classification criterion for the intensity of posterior pharyngeal wall compression has been suggested. On this one, it is considered mild compression when the reduction of the pharyngeal lumen is up to 30%, moderate when this reduction is between 30% and 50%, and severe if the reduction exceeds 50% of the pharyngeal lumen^(11)^.

Other symptoms associated with ACO include airway obstruction, dysphonia and odynophagia. Dyspnea can be explained not only by the mechanical obstacle in the airways, but also by the retrocricoid inflammation generated by osteophytes, which causes a reduction in glottic mobility. This mechanism also explains the presence of dysphonia and stridor. Osteophytes at the level of C2 and C3 generate a greater risk of airway compromise^(9)^.

Before defining the osteophyte as the etiopathogenic factor of dysphagia, the most common causes of dysphagia in the elderly population should be researched and excluded, such as presbyphagia, stroke, dementia, neurodegenerative diseases, tumor lesions, motility disorders, sarcopenia, achalasia, strictures and use of medications. For OCA diagnosis, imaging investigation can be performed with lateral cervical radiography or CT, which is more accurate and provides an image with better definition of soft tissues, essential if surgery is indicated^(12)^. The differential diagnosis of the lesion should include submucosal pharyngeal tumors, cervical dislocation and comminuted fracture of the cervical vertebra.

Functionally, dysphagia secondary to ACO can be assessed with FEES or videofluoroscopy. In both exams, posterior pharyngoesophageal compression, cricopharyngeal spasm, nasal reflux of food or residues on the lesion can be observed, especially in solid consistency^(11)^. The FEES, in addition to clarifying the dynamics of swallowing and the efficiency of the pharyngeal phase, identifies anatomical and/or functional alterations in the structures. In the case of cervical osteophytes, a bulging, submucosal and regular lesion is directly seen on the posterior pharyngeal wall. There may also be a reduction in laryngeal elevation, penetration and difficulty in retroversion of the epiglottis due to the anatomical barrier imposed by the osteophyte^(11)^.

The initial treatment strategy for symptomatic OCA should be conservative. There is usually a good response to speech therapy for swallowing, through the adequacy of the consistency of the diet and postural guidance, such as swallowing with cervical flexion or the chin tuck maneuver. Medications such as muscle relaxants, proton pump inhibitors and, if there is significant tissue inflammation, corticosteroids may be prescribed^(9)^.

The specific surgical treatment for anterior cervical osteophytes is osteophytectomy, which is considered to be highly effective. There are no scientific recommendations that clearly define when surgery should be indicated, but there is evidence of benefit in the event of failure of conservative treatment - defined by persistent dysphagia with involuntary weight loss despite initial measures - or in the presence of airway obstruction higher. Typically, the symptoms must be associated with osteophytes larger than 10 mm, which would be enough to establish a cause-effect relationship and attribute the actual clinical picture to the lesion. It is estimated that 8 to 10% of patients with dysphagia secondary to ACO require surgical treatment^(12)^.

The surgical approach to cervical osteophytes depends on their location. Higher lesions (C1-C2) are resectable via the trans-oral or transcervical route, while osteophytes located in the middle or lower cervical spine (C3-C7) have the only possibility of approaching the transcervical anterolateral route^(13)^. Significant symptomatic improvement is observed within 3 months after surgery. A good prognostic factor for osteophytectomy is the limitation to opening the UES by the osteophyte in the preoperative period, as it speaks in favor of a mechanical cause for dysphagia and, therefore, reversible with the removal of the lesion^(13)^. Persistent dysphagia after surgery is often due to irreversible inflammatory changes in the tissue adjacent to the osteophyte, caused by the long and progressive history of osteophytogenesis^(6)^.

Surgery complications include hematomas and damage to the superior laryngeal, recurrent laryngeal, and hypoglossal nerves. Recurrence of the lesion may also occur after surgery, with a very variable frequency in the literature, with rates ranging from 0 to 100% in follow-ups from 17 months to 13 years^(7)^.

## FINAL COMMENTS

Anterior cervical osteophytes are common in the elderly population. Although they are rarely involved in the etiology of dysphagia, considering its high prevalence, they should be part of the systematic investigation of dysphagia in the elderly. Diagnosis is performed with computed tomography of the cervical spine and FEES. Symptoms respond well to conservative treatment with speech therapy for swallowing. It is important that otorhinolaryngologists and speech therapists are aware of this diagnosis, allowing for an early and effective treatment for the assisted patient.

## References

[1] de Lima Alvarenga EH, Dall’Oglio GP, Murano EZ, Abrahão M (2018). Continuum theory: presbyphagia to dysphagia? Functional assessment of swallowing in the elderly. Eur Arch Otorhinolaryngol.

[2] Ortega O, Martín A, Clavé P (2017). Diagnosis and management of oropharyngeal dysphagia among older persons, state of the art. J Am Med Dir Assoc.

[3] Baijens LWJ, Clavé P, Cras P, Ekberg O, Forster A, Kolb GF (2016). European society for swallowing disorders - European union geriatric medicine society white paper: oropharyngeal dysphagia as a geriatric syndrome. Clin Interv Aging.

[4] Shimizu M, Kobayashi T, Jimbo S, Senoo I, Ito H (2018). Clinical evaluation of surgery for osteophyte-associated dysphagia using the functional outcome swallowing scale. PLoS One.

[5] Verlaan JJ, Boswijk PFE, De Ru JA, Dhert WJA, Oner FC (2011). Diffuse idiopathic skeletal hyperostosis of the cervical spine: an underestimated cause of dysphagia and airway obstruction. Spine J.

[6] Lui Jonathan YC, Sayal P, Prezerakos G, Russo V, Choi D, Casey ATH (2018). The surgical management of dysphagia secondary to diffuse idiopathic skeletal hyperostosis. Clin Neurol Neurosurg.

[7] Sebaaly A, Boubez G, Sunna T, Wang Z, Alam E, Christopoulos A (2018). Diffuse Idiopathic hyperostosis manifesting as dysphagia and bilateral cord paralysis: a case report and literature review. World Neurosurg.

[8] Srivastava SK, Bhosale SK, Lohiya TA, Aggarwal RA (2016). Giant cervical osteophyte: an unusual cause of dysphagia. J Clin Diagn Res.

[9] Seidler TO, Pèrez Àlvarez JC, Wonneberger K, Hacki T (2009). Dysphagia caused by ventral osteophytes of the cervical spine: clinical and radiographic findings. Eur Arch Otorhinolaryngol.

[10] Strasser G, Schima W, Schober E, Pokieser P, Kaider A, Denk DM (2000). Cervical osteophytes impinging on the pharynx: importance of size and concurrent disorders for development of aspiration. AJR Am J Roentgenol.

[11] Choi HE, Jo GY, Kim WJ (2019). Characteristics and clinical course of dysphagia caused by anterior cervical osteophyte. Ann Rehabil Med.

[12] Ruetten S, Baraliakos X, Godolias G, Komp M (2019). Surgical treatment of anterior cervical osteophytes causing dysphagia. J Orthop Surg (Hong Kong).

[13] Chung YS, Zhang HY, Ha Y, Park JY (2020). Surgical outcomes of dysphagia provoked by diffuse idiopathic skeletal hyperostosis in the cervical spine. Yonsei Med J.

