# Supplementary material for: Dysphagia due to anterior cervical osteophytosis: case report
Source: Codas. 2021 Dec 20;34(2):e20200435. doi: 10.1590/2317-1782/20212020435 (PMC9927700; doi:10.1590/2317-1782/20212020435)
Supplement: Translation

# Translation: Disfagia por osteofitose cervical anterior: relato de caso

**Authors:** Mateus Morais Aires, Gabriela Marie Fukumoto, Sarah Lima Ribeiro, Leonardo Haddad, Eliézia Helena de Lima Alvarenga

**Affiliations:** 1 Departamento de Otorrinolaringologia e Cirurgia de Cabeça e Pescoço, Escola Paulista de Medicina, Universidade Federal de São Paulo – UNIFESP – São Paulo (SP), Brasil.; 2 Faculdade de Medicina de Ribeirão Preto, Universidade de São Paulo – USP – Ribeirão Preto (SP), Brasil.

**Keywords:** Transtornos de deglutição, Osteófito, Osteofitose espinhal, Envelhecido, Terapia de fala

## Abstract

Osteofitose cervical anterior é uma condição não inflamatória caracterizada por calcificação ou ossificação dos ligamentos paravertebrais anterolaterais da coluna cervical. Acomete 20 a 30% dos idosos, sendo responsável por 1,6% das etiologias identificáveis da disfagia na população senil. Em estados avançados, a disfagia por osteofitose cervical pode levar a complicações como desnutrição, perda de peso e pneumonia aspirativa. Este estudo visa alertar para a suspeição desse diagnóstico à equipe multidisciplinar que cuida do idoso disfágico, possibilitando tratamento oportuno e precoce da condição. É relatado o caso de um paciente masculino de 66 anos com disfagia tipo engasgo para sólidos e refluxo nasal de alimentos há 1 ano. Videoendoscopia da deglutição evidenciou abaulamento da parede posterior da faringe e, à oferta de alimento sólido, restrição à retroflexão da epiglote, refluxo nasal do alimento e grande quantidade de resíduo alimentar sobre a lesão. Tomografia computadorizada de coluna cervical identificou a presença de osteófitos cervicais anteriores entre as vértebras C3 e C6, o maior com comprimento anteroposterior de 12 milímetros, estreitando a coluna aérea ao nível da oro- e hipofaringe. O paciente foi adequadamente tratado com fonoterapia da deglutição. A estratégia inicial de tratamento para a osteofitose sintomática deve ser conservadora, geralmente com boa resposta à fonoterapia da deglutição. Apesar de raramente estarem implicados na etiologia da disfagia, considerando sua alta prevalência, é importante que otorrinolaringologistas e fonoaudiólogos estejam atentos a esse diagnóstico, permitindo tratamento precoce e efetivo para o paciente assistido, melhor prognóstico e menos complicações da disfagia orofaríngea no idoso.

## INTRODUÇÃO

A deglutição é um processo neuromuscular sinérgico, sequencial e harmônico, em que o alimento é transportado da boca até o estômago. Qualquer disrupção no movimento de deglutição, antes, durante ou após a alimentação, pode ser definido como disfagia^(1)^. A presbifagia, por sua vez, é entendida como alteração da deglutição em idosos saudáveis e pode ser silenciosa e lentamente progressiva, pois ocorre paralelamente ao processo de envelhecimento. Pode estar associada a alterações naturais da anatomia da cabeça e pescoço e ao declínio gradativo das funções fisiológicas e neurológicas, com diminuição da elevação laríngea, da abertura do esfíncter superior esofágico e dos movimentos peristálticos^(2)^. Uma questão preocupante ocorre quando o estado de saúde do idoso é prejudicado por algum fator agressor, acarretando perda do seu delicado equilíbrio e dos mecanismos que compensam o distúrbio da deglutição, desenvolvendo assim a disfagia. Estima-se que a prevalência de disfagia em maiores de 60 anos seja de 27%^(3)^.

A perda da reserva funcional (capacidade resiliente do corpo de se adaptar ao estresse fisiológico) torna a população idosa mais suscetível à disfagia^(1)^. Comorbidades e doenças crônicas, uso de medicações, comprometimento cognitivo após eventos cerebrovasculares ou doenças do aparelho aerodigestivo superior, que são mais prevalentes entre os idosos quando comparados aos indivíduos mais jovens, podem prejudicar a função presbifágica bem adaptada e levar à disfagia e aspiração^(1)^.

Cada vez mais, desordens da deglutição têm sido reconhecidos como problemas emergentes por profissionais de saúde, especialmente os que lidam com a população senil. Recentemente, a *European Society for Swallowing Disorders* e a *European Union Geriatric Medicine Society* reconheceram a disfagia como uma síndrome geriátrica^(3)^. Suas principais complicações são desnutrição, desidratação e afecções respiratórias como pneumonia aspirativa. Adicionalmente, vários estudos têm destacado o impacto negativo social e psicológico da disfagia, como desprazer à alimentação, ansiedade, pânico e isolamento social, em incidência tão expressiva quanto em 55% dos idosos institucionalizados^(1,3)^.

﻿﻿O tratamento sindrômico da disfagia orofaríngea no idoso é baseado em medidas compensatórias, como adaptação da consistência do alimento, correção da postura e manobras de deglutição. Se uma causa específica de disfagia for identificada, o tratamento etiológico direcionado pode ser realizado, com melhor prognóstico para resolução da disfagia e resgate da qualidade de vida^(2)^.

Osteofitose cervical anterior (OCA) é uma condição não inflamatória caracterizada por ﻿calcificação ou ossificação ao longo de ligamentos paravertebrais anterolaterais da coluna cervical. Osteófitos cervicais são comuns nos idosos, com prevalência de 20 a 30%, e podem ser uma causa de disfagia nessa população﻿. Geralmente são achados radiológicos assintomáticos ou provocam sintomas pouco específicos, como limitação do movimento cervical ou dor local. Caso sejam mais volumosos, podem chegar a comprimir a parede posterior da faringe ou do esôfago, levando à disfagia orofaríngea. Em estados avançados, o idoso pode apresentar desnutrição e perda de peso devido à disfagia causada pelos osteófitos cervicais^(4)^.

﻿Estima-se que a OCA seja responsável por 1,6% das etiologias identificáveis da disfagia do idoso^(5)^. Apesar de ser uma rara causa, é importante que seja pesquisada, já que é uma etiologia potencialmente curável com tratamento adequado, seja conservador ou cirúrgico. Quanto mais precoce o tratamento, melhores os resultados, pois há menos alterações teciduais irreversíveis causadas pela exposição crônica aos osteófitos no trato digestivo alto, como inflamação e fibrose com diminuição da peristalse^(6)^.

Uma vez que há poucos casos de disfagia secundária à OCA relatados na literatura, este estudo visa alertar para a suspeição desse diagnóstico à equipe multidisciplinar que cuida do idoso disfágico. Com isso, possibilitaria tratamento precoce da condição, melhor prognóstico e menor incidência de complicações da disfagia orofaríngea no idoso.

## APRESENTAÇÃO DO CASO CLÍNICO

O paciente apresentado assinou o Termo de Consentimento Livre e Esclarecido e os demais princípios éticos foram cumpridos, respeitando a Resolução 466/12 do Conselho Nacional de Ética em Pesquisa. Este relato de caso foi aprovado pelo Comitê de Ética em Pesquisa da Universidade Federal de São Paulo (UNIFESP), sob parecer número ﻿4.455.365.

Paciente masculino de 66 anos compareceu à consulta otorrinolaringológica com queixa de disfagia tipo engasgo para sólidos e refluxo nasal de alimentos há 1 ano. Sofreu acidente automobilístico há 13 anos e apresenta diabetes melitus. Realizada videoendoscopia da deglutição (VED), sendo visualizado na avaliação estrutural abaulamento da parede posterior da hipofaringe, com resíduo salivar sobre a lesão (Figura 1). Na avaliação funcional, após oferta de alimento sólido, observou-se restrição à retroflexão da epiglote, limitação da elevação da laringe e refluxo nasal do alimento, com grande quantidade de resíduo alimentar acima da lesão, na parede posterior da faringe. No teste de manobras posturais, houve piora da disfagia à extensão cervical e melhor *clearance* à flexão da cabeça. Tomografia computadorizada (TC) de coluna cervical, solicitada para avaliação da natureza da lesão, evidenciou presença de osteófitos cervicais anteriores entre as vértebras C3 e C6, o maior com comprimento anteroposterior de 12 milímetros (mm), estreitando a coluna aérea ao nível da oro- e hipofaringe (Figura 2). Foram excluídas outras causas de disfagia e o paciente foi tratado com fonoterapia da deglutição e omeprazol, este direcionado ao refluxo faringolaríngeo. Foram realizadas 6 sessões de fonoterapia semanal individualizada, com o objetivo de compensar o mecanismo da deglutição diante de um obstáculo mecânico na região faríngea. Os principais aspectos trabalhados foram: modificação da textura da dieta, evitando-se alimentos sólidos e secos; manobras posturais de deglutição, testadas durante a VED, especialmente o *chin tuck* (“cabeça para baixo” ou “flexão da cabeça”), que aumenta o espaço da valécula (“abre a valécula”) e melhora a pressão de transferência faríngea do alimento; e exercícios de fortalecimento da língua, para transpor a barreira mecânica da orofaringe durante a ejeção do alimento. Recomendou-se ainda, se necessário, diante da percepção de alimento estagnado na faringe, alternar a oferta de alimentos sólidos com líquidos. Foi orientada repetição dos exercícios de língua em domicílio, 2 a 3 vezes ao dia. Após as sessões iniciais, o paciente apresentou resposta satisfatória (Figura 3), mantendo acompanhamento trimestral no primeiro ano e semestral a partir do segundo ano. Encaminhado para avaliação ortopédica para discutir viabilidade cirúrgica, optou-se por tratamento conservador devido à boa compensação com medidas clínicas. Segue em acompanhamento ambulatorial há 2 anos, mantendo deglutição eficiente, sem incidência de complicações relacionadas à disfagia. Optou-se por manter reavaliações periódicas, ao menos uma vez ao ano, para detecção precoce de possível novo desequilíbrio da homeostase da deglutição relacionado ao declínio gradativo das funções no contexto da presbifagia ou ao surgimento de um novo fator agressor.

**Figura 1:** Videoendoscopia da deglutição. Avaliação estrutural demonstrando (A) abaulamento esférico da parede posterior da orofaringe, pronunciando-se anteriormente em direção ao palato mole e base da língua e estreitando a via aerodigestiva. Avaliação funcional com consistência sólida evidenciando (B) refluxo nasal de alimento e (C) *clearance* ineficaz com resíduo alimentar sobre a lesão após 3 deglutições

**Figura 2:** Tomografia computadorizada de pescoço, cortes sagital (A) e axial (B), evidenciando osteófitos cervicais de C3 a C6, o maior (seta) medindo 12mm no diâmetro anteroposterior

**Figura 3:** Videoendoscopia da deglutição após fonoterapia. Avaliação funcional com manobra *chin tuck*, (A) antes da deglutição, evidenciando aumento do espaço faríngeo e valecular pela manobra, e (B) durante a deglutição, com diminuição do refluxo nasal de alimento

## DISCUSSÃO

Osteófitos cervicais anteriores são um achado radiológico comum na população idosa, acometendo 20 a 30% dos pacientes acima de 60 anos^(7)^. Entre suas possíveis etiologias, destacam-se hiperostose esquelética difusa idiopática (doença de Forestier), espondilite anquilosante, doença degenerativa da coluna cervical e reparação óssea após trauma. Além da idade avançada, sexo masculino (6:1 em relação ao feminino) e obesidade são fatores de risco bem estabelecidos para o desenvolvimento de osteófitos^(5)^. De acordo com revisão sistemática conduzida por Verlaan et al.^(5)^, o local mais comum de acometimento do osteófito é entre as vértebras C3 e C5. As excrescências ósseas costumam variar de 1 a 2 mm, mas podem chegar a 30 mm. O paciente relatado é um típico portador de osteófito, uma vez que é do sexo masculino, idoso, possui histórico de trauma e tem acometimento nas vértebras C3 a C6.

Osteófitos cervicais, mesmo volumosos, são frequentemente assintomáticos^(8)^. A hiperostose crônica parece ser relativamente bem tolerada até que um “evento gatilho”, como asfixia, aspiração ou trauma cervical menor, promova um desbalanço na reserva compensatória já limítrofe, levando ao surgimento de um quadro clínico. Múltiplos mecanismos fisiopatológicos têm sido descritos para explicar a sintomatologia relacionada aos osteófitos, como^(1)^ efeito de massa direto, causando obstrução mecânica ou desvio da faringe ou esôfago^(2)^; ancoragem do esfíncter esofagiano superior (EES) ao nível da cartilagem cricoide^(3)^; restrição ao movimento da laringe ou epiglote^(4)^; inflamação do tecido mole subjacente, levando a fibrose e estenose; e^(5)^ neuropatia inflamatória^(9)^.

Os sintomas mais comuns são redução da extensão do movimento cervical e dor local. OCA raramente causa disfagia. Em uma série de 116 pacientes com OCA, apenas 7 (6%) apresentavam disfagia^(7)^. Estudos mostram que a incidência de disfagia secundária a OCA varia de 0,1 a 33%, dependendo da definição de disfagia e do método diagnóstico^(7)^. Por outro lado, raramente a disfagia tem como causa osteófitos cervicais. Em uma série de 3318 pacientes com disfagia, apenas 55 (1,6%) tinham osteófitos, sendo que mais da metade desses possuía concomitantemente outra etiologia contribuinte à disfagia^(10)^.

﻿ Osteófitos provenientes da quinta e sexta vértebras cervicais (C5 e C6) são os mais implicados em causar disfagia. Não existe uma correlação linear entre tamanho do osteófito e sintomatologia, mas se sabe que osteófitos maiores de 10mm aumentam significantemente o risco de aspiração^(11)^. Uma coorte recente com 10 pacientes portadores de OCA candidatos a tratamento cirúrgico mostrou que metade apresentava perda de peso e desnutrição devido à disfagia, ratificando que o quadro clínico pode ser grave^(4)^. Foi sugerido um critério de classificação para a intensidade da compressão da parede posterior da faringe. Neste, considera-se leve compressão quando a redução do lúmen faríngeo é de até 30%, moderada quando esta redução está entre 30% e 50%, e grave se a redução ultrapassa 50% do lúmen faríngeo^(11)^.

Outros sintomas associados à OCA incluem obstrução de via aérea, disfonia e odinofagia. ﻿A dispneia pode ser explicada não apenas pelo obstáculo mecânico nas vias aéreas, mas também pela inflamação retrocricoide gerada por osteófitos, a qual gera redução da mobilidade glótica. Esse mecanismo explica ainda a presença de disfonia e estridor. Osteófitos em nível de C2 e C3 geram maior risco de comprometimento da via aérea^(9)^.

Antes de se definir o osteófito como o fator etiopatogênico da disfagia, deve-se pesquisar e excluir causas mais comuns de disfagia na população idosa, como presbifagia, acidente vascular encefálico, demência, doenças neurodegenerativas, lesões tumorais, desordens de motilidade, sarcopenia, acalásia, estenoses e uso de medicações. Para diagnóstico da OCA, a investigação por imagem pode ser feita com radiografia cervical lateral ou TC, sendo que esta é mais acurada e fornece imagem com melhor definição de tecidos moles, sendo essencial caso a cirurgia seja indicada^(12)^. O diagnóstico diferencial da lesão deve incluir tumores submucosos de faringe, luxação cervical e fratura cominutiva de vértebra cervical.

Funcionalmente, a disfagia secundária à OCA pode ser avaliada com VED ou videodeglutograma. Em ambos os exames, pode-se observar compressão faringoesofágica posterior, espasmo cricofaríngeo, refluxo nasal de alimento ou resíduos sobre a lesão, especialmente na consistência sólida^(11)^. A VED, além de esclarecer a dinâmica da deglutição e a eficiência da fase faríngea, identifica alterações anatômicas e/ou funcionais das estruturas. No caso dos osteófitos cervicais, vê-se de forma direta uma lesão abaulada, submucosa e regular na parede posterior da faringe. Pode haver ainda diminuição da elevação laríngea, penetração e dificuldade de retroversão da epiglote devido à barreira anatômica imposta pelo osteófito^(11)^.

A estratégia inicial de tratamento para a OCA sintomática deve ser conservadora. Geralmente há boa resposta à fonoterapia da deglutição, por meio de adequação da consistência da dieta e orientação postural, como deglutição com flexão cervical ou manobra *chin tuck*. Podem ser prescritas medicações como relaxantes musculares, inibidores de bomba de prótons e, caso haja inflamação tecidual importante, corticoesteroides^(9)^.

O tratamento cirúrgico específico para os osteófitos cervicais anteriores é a osteofitectomia, considerada altamente efetiva. Não há recomendações científicas que definam claramente quando a cirurgia deve ser indicada, mas há evidência de benefício no caso de falha do tratamento conservador – definida por persistência da disfagia com perda involuntária de peso apesar das medidas iniciais – ou em vigência de obstrução de via aérea superior. Tipicamente, a sintomatologia deve estar associada a osteófitos maiores que 10 mm, o que seria suficiente para estabelecer uma relação de causa-efeito e atribuir o quadro clínico de fato à lesão. Estima-se que 8 a 10% dos pacientes com disfagia secundária à OCA necessitem de tratamento cirúrgico^(12)^.

A via de abordagem cirúrgica dos osteófitos cervicais depende da localização. Lesões mais altas (C1-C2) são ressecáveis via trans-oral ou transcervical, enquanto osteófitos localizados na coluna cervical média ou baixa (C3-C7) têm como única possibilidade a abordagem via transcervical anterolateral^(13)^. Observa-se melhora sintomática significativa em até 3 meses após a cirurgia. Um fator de bom prognóstico para a osteofitectomia é a limitação à abertura do EES pelo osteófito no pré-operatório, porque fala a favor de causa mecânica para a disfagia e, assim, reversível com a exérese da lesão^(13)^. Disfagia persistente após a cirurgia deve-se frequentemente a alterações inflamatórias irreversíveis no tecido adjacente ao osteófito, causadas pela história longa e progressiva de osteofitogênese^(6)^.

Complicações da cirurgia incluem hematoma e lesão dos nervos laríngeo superior, laríngeo recorrente e hipoglosso. Pode ocorrer ainda recorrência da lesão após a cirurgia, com frequência bastante variável na literatura, com taxas de 0 a 100% em seguimentos de 17 meses a 13 anos^(7)^.

## COMENTÁRIOS FINAIS

Osteófitos cervicais anteriores são comuns na população idosa. Apesar de raramente estarem implicados na etiologia da disfagia, considerando sua alta prevalência, devem fazer parte da investigação sistemática da disfagia nos idosos. O diagnóstico é realizado com tomografia computadorizada de coluna cervical e videoendoscopia da deglutição. A sintomatologia apresenta boa resposta ao tratamento conservador com fonoterapia da deglutição. É importante que otorrinolaringologistas e fonoaudiólogos estejam atentos a esse diagnóstico, permitindo tratamento precoce e efetivo para o paciente assistido.
